# HIV-1/Cocaine Induced Oxidative Stress Disrupts Tight Junction Protein-1 in Human Pulmonary Microvascular Endothelial Cells: Role of Ras/ERK1/2 Pathway

**DOI:** 10.1371/journal.pone.0085246

**Published:** 2014-01-07

**Authors:** Pranjali Dalvi, Kun Wang, Joel Mermis, Ruoxi Zeng, Miles Sanderson, Sara Johnson, Yuqiao Dai, Garima Sharma, Amy O’Brien Ladner, Navneet K. Dhillon

**Affiliations:** 1 Division of Pulmonary and Critical Care Medicine, University of Kansas Medical Center, Kansas City, Kansas, United States of America; 2 Department of Molecular and Integrative Physiology, University of Kansas Medical Center, Kansas City, Kansas, United States of America; University of Louisville, United States of America

## Abstract

Intravenous drug use (IVDU) is the major risk factor in the development of HIV-related pulmonary arterial hypertension (HRPAH); however, the pathogenesis of HRPAH in association with IVDU has yet to be characterized. Endothelial injury is considered to be an initiating factor for pulmonary vascular remodeling in animal models of PAH. Our previous study shows that simultaneous exposure to HIV-Trans-activator of transcription (Tat) and cocaine exacerbates both disruption of tight junction proteins and permeability of human pulmonary artery endothelial cells compared with either treatment alone. We here now demonstrate that this HIV-Tat and cocaine mediated endothelial dysfunction accompanies with increase in hydrogen peroxide and superoxide radicals generation and involves redox sensitive signaling pathway. Pretreatment with antioxidant cocktail attenuated the cocaine and Tat mediated disassembly of Zonula Occludens (ZO)-1 and enhancement of endothelial monolayer permeability. Furthermore, inhibition of NADPH oxidase by apocynin or siRNA-mediated knockdown of gp-91^phox^ abolished the Tat/cocaine-induced reactive oxygen species (ROS) production, suggesting the NADPH oxidase mediated generation of oxidative radicals. In addition, ROS dependent activation of Ras and ERK1/2 Kinase was observed to be mediating the TJP-1 disassembly, and endothelial dysfunction in response to cocaine and Tat exposure. In conclusion, our findings demonstrate that Tat/cocaine -mediated production of ROS activate Ras/Raf/ERK_1/2_ pathway that contributes to disruption of tight junction protein leading to pulmonary endothelial dysfunction associated with pulmonary vascular remodeling.

## Introduction

HIV-related pulmonary arterial hypertension (HRPAH) is a devastating non-infectious complication associated with HIV-1 infection [1], [2]. PAH associated with HIV-infection has higher mortality compared to other forms of PAH. Furthermore, intravenous drug use (IVDU) has been found to be one of the major risk factors in the development of HRPAH [1], [3]. A range of 59 to 70% of HRPAH cases are reported to be in individuals who also use intravenous drugs [1], [3]. It is evident from various case reports that abuse of cocaine and other stimulants is a possible risk factor in the development of PAH [4]. This is particularly concerning, as cocaine is the second most commonly used illicit drug in the United States [5] and is associated with high blood pressure, vasoconstriction and atherosclerosis [6]. By examining the post-mortem lung sections of patients with HIV-infection and a history of IV drug use, we earlier demonstrated that cocaine and/or opioid use contributes to enhanced HIV-1 related pulmonary vascular remodeling [7].

The pathology of HRPAH is complex. Based on animal studies, it is believed that endothelial dysfunction is the initiating factor of vascular remodeling followed by pulmonary vascular smooth muscle cell proliferation leading to medial hypertrophy [8]. Direct HIV-infection of pulmonary vascular endothelial and smooth muscle cells is not the inciting insult leading to HRPAH development as there is no evidence of HIV-1 RNA or DNA in the pulmonary vessels of human lung tissues [9]. Studies on macaques infected with simian immunodeficiency virus (SIV) or SHIV have also suggested the presence of pulmonary arteriopathy similar to that seen in HIV-infected individuals with HRPAH [10], [11], but without any evidence of the virus or viral DNA in pulmonary endothelial or smooth muscle cells [10], [12]. Recent research now indicates that HIV-1 protein mediated effects rather than direct HIV-1 infection may be initiating the endothelial injury. For example, we recently demonstrated the development of pulmonary vascular remodeling under the influence of HIV-1 proteins alone, without viral infection, [13] in a non-infectious HIV-transgenic rat model.

The HIV-1 protein, Trans- activator of transcription (Tat), is actively secreted by infected cells and has been detected in the serum of HIV-infected patients [14]. HIV-Tat can therefore, elicit responses in various other target cells by either easy entry in to the cells or interacting with cell-surface receptors. Tat is known to bind Flk-1/KDR, a vascular endothelial growth factor receptor-2 (VEGFR-2) [15] or integrins [16] and act as an angiogenic and oncogenic factor [17] by promoting growth, migration and production of growth factors in various cell-types [17]. Our earlier studies demonstrate that HIV-proteins including Tat induce reactive oxygen species (ROS) in pulmonary endothelial cells and consequently activate platelet-derived growth factor, a critical mediator implicated in the pathogenesis of HRPAH [13].

In our previous findings, HIV-infected cocaine and/or opioid users showing signs of pulmonary arteriopathy had significantly decreased expression of tight junction proteins (TJPs) compared to lungs from HIV-infected individuals without the history of IVDU [7]. In addition, we also showed *in-vitro* that combined treatment with Tat and cocaine increases pulmonary endothelial cell permeability with decrease in Zonula Occludens (ZO)-1 or TJP-1 expression at the periphery [7]. It is likely that HIV-proteins interact with cocaine and together accelerate the development of pulmonary vascular endothelial dysfunction. Given that ROS significantly contributes to endothelial dysfunction [18] and; both Tat [19] and cocaine [20] are known to induce oxidative stress, we hypothesize that Tat and cocaine mediated enhanced disruption of TJPs involves increased oxidative stress and modulation of downstream redox-sensitive signaling pathway in the pulmonary endothelium.

## Materials and Methods

### Materials

Cocaine hydrochloride, SU5416 (antagonist of VEGFR-2), and BD1047 (antagonist of sigma receptor) was obtained from Sigma Aldrich (St. Louis, MO). HIV-1 Tat 1–72 was purchased from University of Kentucky College of Medicine (Lexington, KY). U0126, phosphorylated ERK (Thr202/Tyr204) and PCNA antibody were purchased from Cell Signaling Technology (Beverly, MA). Glutathione, α-tocapherol and β-integrin antibody were purchased from Santa Cruz Biotechnology, Inc (Santa Cruz, CA). L-ascorbate acid sodium salt was obtained from ACROS Organics (Belgium). ZO-1 antibody for immunocytofluorescence staining was purchased from Life Technologies (Carlsbad, CA). ZO-1 antibody for Western blot, Ras activation assay kit, *In- vitro* vascular permeability assay kit, and compartmental protein extraction kit were purchased from EMD Millipore (Billerica, MA).

### Cell Culture and Treatments

Human Pulmonary Microvascular Endothelial Cells (HPMECs) were purchased for ScienCells Research Laboratory (Carlsbad, CA) and were cultured in endothelial cell medium supplemented with 5% FBS, 1% endothelial cell growth factor, and penicillin (100 IU/ml )/streptomycin (50 µg/ml). Cells were treated with 1 µM cocaine and/or 25 ng/ml Tat and concentrations used was based on our previous publication [7]. HPMECs were pre-treated with NADPH oxidase inhibitor (apocynin, 25 µM and 250 µM), VEGFR inhibitor (SU5416, 0.1 µM), sigma receptor inhibitor (BD1047, 10 nM), catalase (200 U/ml), superoxide dismutase (300 U/ml), MEK1/2 inhibitor (U0126, 10 µM), or antioxidant cocktail (0.2 mM ascorbate, 0.5 mM glutathione, and 3.5 µM tocopherol) for 5 to 30 min followed by cocaine/Tat treatment for 1 hour for ROS, H_2_O_2_, and superoxide radical assays or 6 to 24 h for endothelial permeability and Western blot analysis.

### Quantification of Cellular Oxidative Stress

To measure total amount of intracellular reactive oxygen species, pulmonary endothelial cells were plated onto 96-well plate (2.0×10^4^cell/well) and cultured for 2 days. Cells were washed with serum free medium and incubated with 15 µM 5-(and -6)-carboxy-2′, 7′-dichlorodihydroflourescein diacetate (DCFH-DA) (Molecular Probes, Inc.) for 30 min followed by incubation with apocynin (NOX inhibitor 25 or 250 µM) for 10 min before cocaine (1 µM) and/or Tat (25 ng/ml) exposure for 15, 30, 60, and 90 min. In the presence of ROS_,_ DCFH is oxidized to a fluorescent DCF within the cytoplasm which was read by fluorescent plate reader at an excitation of 485 nm with an emission of 530 nm.

Production of H_2_O_2_ was measured by Amplex red assay kit (Invitrogen, A-22188, Carlsbad, CA). HPMECs seeded on 96 well plates were exposed to cocaine (1 µM) and/or Tat (25 ng/ml) in presence or absence of catalase (Sigma Aldrich, MO, 10 U/ml) and/or superoxide dismutase (SOD) (Sigma Aldrich, 100U/ml) for 1 h at 37°C. Cells were then rinsed with Krebs-Ringer phosphate buffer (100 µl, pH 7.35) and incubated with pre-warmed 50 µM Amplex Red and 0.1 U/ml HRP prepared in Krebs buffer (100 µl/well). Changes in fluorescence were measured after 10 min on Cytofluor multi well plate reader (PerSeptive Biosystems). Amplex red assay solution with or without catalase and/or SOD incubated in absence of cells for 10 min was obtained as basal value for the respective treatments. Hydrogen peroxide production, calculated based on the standard curve, was normalized according to the number of cells per well by Cyquant assay kit (Invitrogen, C7026). Data are represented as µM/min/10^4^ cells.

Superoxide radicals (O_2_^−^) generation was examined using superoxide dismutase (SOD) inhibitable cytochrome-c reduction assay. Briefly, HPMECs grown on 96 well plates were incubated with cytochrome c (Sigma Aldrich, 20 µM, 100 µl/well prepared in phenol red free OptiMEM medium, Invitrogen) containing 1 µM cocaine and/or Tat (25 ng/ml), with or without SOD (100U/ml). The absorbance of the medium was read at 550 nm after 30 min of incubation. The release of O_2_^−^ was calculated using the equation: C =  (Ab_-SOD_–Ab_+SOD_)/(ε×d), where C is the concentration (moles of superoxide), Ab is absorbance in absence and presence of SOD, ε is the extinction coefficient of cytochrome C (21×10^3^ M^−1^cm^−1^) and d is 0.3cm (the vertical light path when 100 µl volumes per well are dispensed in 96-well plates)^1^. O_2_^−^ generation per well was normalized based on the cell number as mentioned above using Cyquant assay kit. The results were expressed as µmoles of O_2_^−^ per min per 10^4^ cells.

### Endothelial Permeability Assay

HPMECs (2.0×10^5^cell/well) were seeded onto collagen-coated transwell inserts (polyethylene membrane, pore size: 1.0 µm) and cultured as monolayer in medium containing 5% FBS for 2 days followed by pre-treatment with antioxidant cocktail for 5 min, or SU5416/BD1047 for 30 min, or catalase/superoxide dismutase for 5 min followed by cocaine/Tat treatment. An *in-vitro* vascular permeability assay (Millipore, Billerica, MA) was performed 6 or 24 hours after Tat/cocaine treatment by measurement of fluorescein isothiocyanate (*FITC*)-dextran permeability across monolayers.

### Transfection of Pulmonary Endothelial Cells with Small Interfering (si) RNA

Small interfering RNA targeting gp91^phox^ (NOX2), a critical subunit of NADPH oxidase in endothelial cells, was used to determine the source of ROS. Silencer select pre-designed and validated siRNA was obtained from Applied Biosystems (Carlsbad, CA). Cells were also transfected with silencer select negative control (scrambled) siRNA for comparison. HPMECs were transfected with 5 nM siRNA using Hiperfect transfection reagent (Qiagen, Valencia, CA) as per manufacturer’s instructions. The transfected cells were then treated with DCFH-DA for 30 min followed by Tat and cocaine for 1 hr for determination of ROS generation as mentioned above. The levels of gp phox91 mRNA were quantitated in transfected or un-transfected cells treated with cocaine and/or Tat by Real-Time RT-PCR using the SYBR Green detection on ABI Prism Fast sequence detector as described previously [13].

### Western Blot Analysis

At the end of treatment, cells were either lysed with RIPA lysis buffer for total protein extract or cytosolic, membrane, and nuclear protein fractions were extracted using compartmental protein extraction kit (Millipore) according to the manufacturer's instructions. Protein extracts were resolved on sodium dodecyl sulfate-polyacrylamide gel (10%) and then electro-transferred to PVDF membranes. Membranes were incubated with primary antibody overnight at 4°C, followed by incubation with secondary antibody and detected by enhanced chemical luminescence kit. Blots were re-probed with β-actin antibody to normalize cytosolic fractions, β-integrin to normalize membrane fractions and PCNA for nuclear compartment. Densitometry analysis using NIH Image J software was performed for quantification of western blots. Experiments were repeated at least three times.

### Ras Pull-down Assay

Activation of Ras (Ras-GTP) was detected using Ras Activation Assay Kit from Millipore (CA, USA) according to the manufacturer’s recommendations. HPMECs were pre-treated with antioxidant cocktails, BD1047, or SU5416 followed by Tat and cocaine treatment. Three hundred microgram of protein was applied for pull-down assay.

### Immunocytofluorescence Staining

HPMECs were seeded onto collagen-coated coverslips (7.5×10^5^cell/well), cultured to confluence and pre-treated with antioxidant cocktails for 5 min, followed by cocaine/Tat treatment for 24 h. Cells were fixed with 4% paraformaldehyde for 30 min at room temperature and incubated overnight with primary antibody (1∶100). Alexa Fluor 488 conjugated secondary antibody (1∶200) and Slow Fade antifade reagent with 4,6-diamidino-2-phenylindole (DAPI) were used to visualize ZO-1 protein and nuclei. Images were captured using a Zeiss LSM510 confocal microscope.

### Statistical Analysis

Statistical analysis was performed using multi-comparison Bonferroni test on STATA12 software. Results were judged statistically significant if p≤0.05.

## Results

### Cocaine and Tat Mediated Enhanced Pulmonary Endothelial Disruption involves Sigma Receptor and VEGFR-2 Binding, Respectively

**Figure 1:**
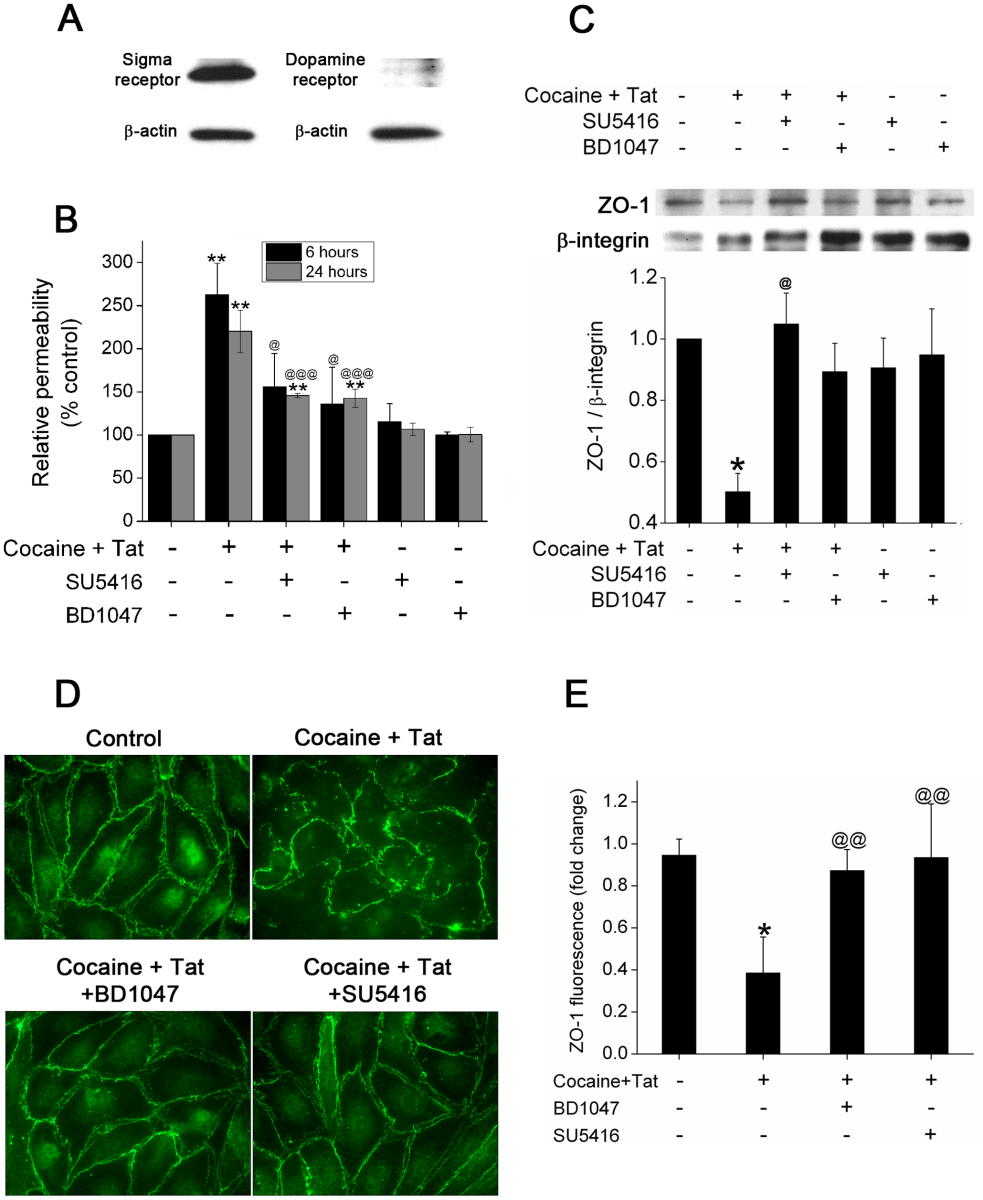
Attenuation of Tat and cocaine mediated endothelial dysfunction in the presence of VEGFR-2 or sigma receptor antagonists. (A) Expression of sigma and dopamine receptors in HPMECs as analyzed by Western blot of total cellular extract. (B) HPMECs were treated with Tat (25 ng/ml) and cocaine (1 µM) for 6 or 24 hours in the presence or absence of SU5416 (antagonist of VEGFR-2) or BD1047 (antagonist of sigma receptor). FITC-Dextran permeability was assessed by using *in-vitro* vascular permeability assay kit. The values shown are means (±SD) of at least three independent experiments. (C) Membrane fraction was isolated and analyzed for ZO-1 by western blot analysis. Blot is representative of at least three independent experiments with histogram showing (lower panel) the average densitometry analysis normalized to β-integrin (mean ± S.E.M). (D) Representative images showing immunocyto-fluorescence staining of ZO-1. (E) Quantification of ZO-1 immunofluorescence using ImageJ software. The values are represented as fold change compared to untreated control. *P≤0.01, **P≤0.001 compared to untreated control; ^@^P≤0.05, ^@@^P≤0.01, ^@@@^P≤0.001 compared to Tat and cocaine treatment.

Our earlier studies reveal that exposure of human pulmonary endothelial cells to both cocaine (1 µM) and Tat (25 ng/ml) results in significant loss of ZO-1(TJP-1) at cell periphery leading to enhanced permeability when compared with either cocaine or Tat treatment alone [7]. We here examined if the effects of Tat and cocaine on the endothelium is specifically through binding to their respective receptors. Cocaine is known to bind both sigma [21] and dopamine [22] receptors, hence, we first analyzed the presence of these receptors in HPMECs. As shown in Figure 1A, western blot analysis of total cellular extract demonstrated abundant presence of sigma receptor while dopamine receptor was sparingly present in HPMECs. Since Tat is known to enter cells by binding to VEGFR-2 [15], [23], we next pre-treated the cells with a potent antagonist of VEGFR2: SU5416 or with antagonist of sigma receptor: BD1047. As illustrated in Figure 1B, pre-treatment with SU5416 or with BD1047 resulted in significant reduction of Tat and cocaine-mediated endothelial permeability. Furthermore down-modulation of ZO-1 protein expression in the cellular membrane of Tat and cocaine treated HPMECs also reversed in presence of SU5416 and BD1047 pre-treatment as observed by western blot analysis of membrane fractions (Figure 1C) or by immunofluorescence (Figure 1D and 1E).

### Enhanced Oxidative Stress in Pulmonary Vascular Endothelial Cells Exposed to Cocaine and Tat

**Figure 2:**
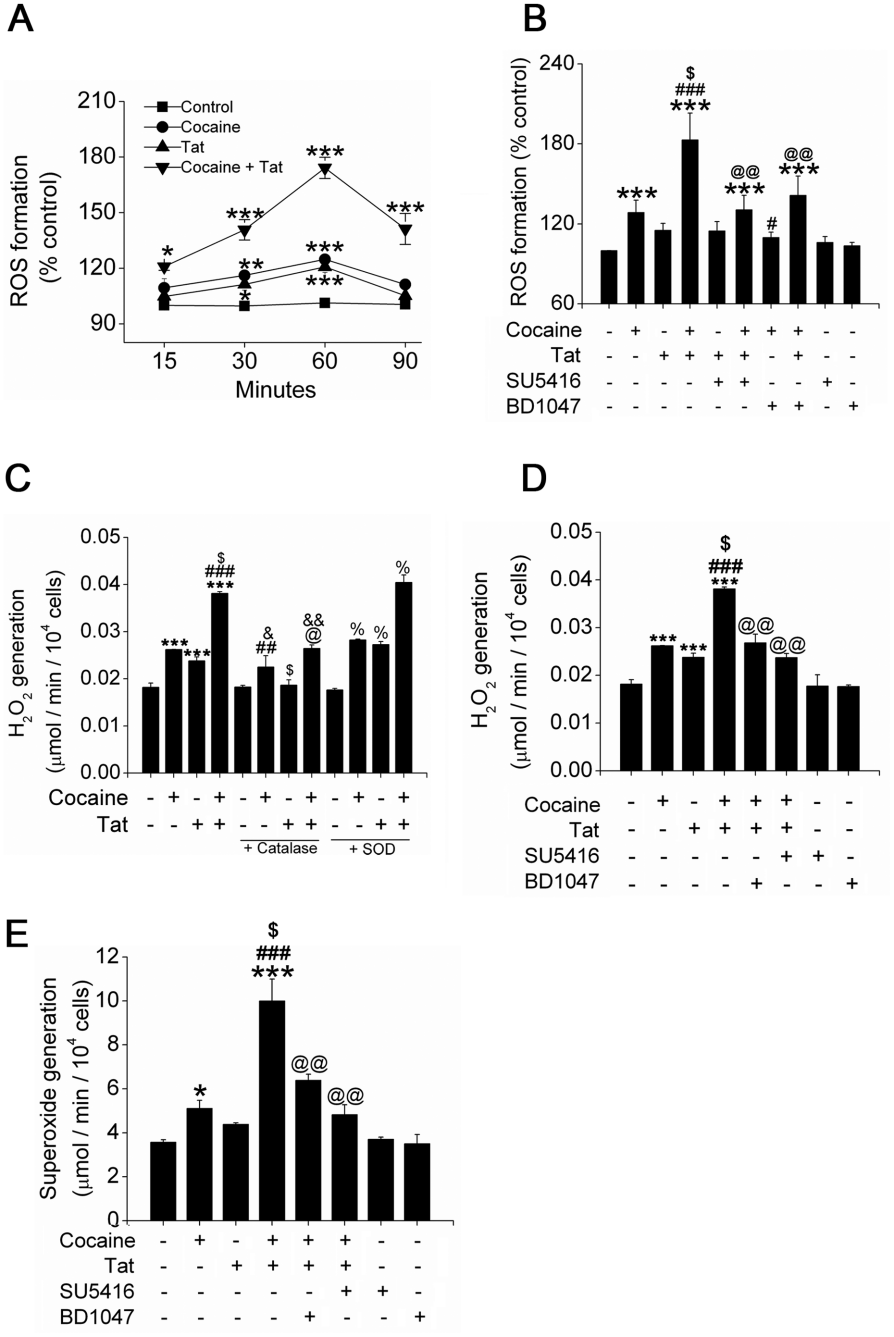
Enhanced oxidative stress on treatment of pulmonary endothelial cells with Tat and cocaine. (A) Generation of ROS in HPMECs treated with Tat and/or cocaine was quantified by DCF assay at indicated time-points. (B) ROS production in cells treated with Tat and cocaine in the presence or absence of SU5416 (antagonist of VEGFR-2) or BD1047 (antagonist of sigma receptor) for 1 hour as analyzed by DCF assay. (C) Generation of H_2_O_2_ was quantified using Amplex red assay kit. HPMECs were treated with Tat and cocaine in the presence or absence of catalase (10U/ml) or SOD (100U/ml) for 1 hour. (D) Reduction of H_2_O_2_ formation on pre-treatment of Tat and cocaine exposed HPMECs with SU5416 or BD1047. (E) Changes in Tat and cocaine-mediated superoxide generation on SU5416 or BD1047 pre-treatment. Formation of superoxide (O_2_^−^) was quantified by SOD-inducible cytochrome c reductase assay. The values shown are means (±SD) of at least three independent experiments. *P≤0.05, **P≤0.01, ***P≤0.001 compared to control; ^#^P≤0.05, ^##^P≤0.01,^ ###^P≤0.001 compared to cocaine treatment; ^$^P≤0.001 compared to Tat treatment;^ @^ P≤0.05, ^@@^ P≤0.001, compared to Tat and cocaine combinational treatment; ^&^P≤0.01, ^&&^P≤0.001 compared to catalase-treated control; ^%^‘P≤0.001, compared to SOD-treated control.

We next evaluated the generation of ROS in cocaine and Tat exposed endothelial cells. As shown in Figure 2A and B, DCFH-DA assay demonstrated significant enhancement in ROS production in HPMECs treated with cocaine and Tat as compared to either cocaine or Tat treatment alone at all the time points tested. Furthermore, blocking of VEGFR-2 or sigma receptor with SU5416 or BD1047 respectively abrogated the generation of Tat or cocaine mediated ROS (Figure 2B). These findings were further confirmed by measurement of hydrogen peroxide (H_2_O_2_) by Amplex red assay (Figure 2C and D), and by measurement of superoxide (O_2_^−^) radicals by cytochrome c reductase assay (Figure 2E). Both amplex red assay and cytochrome c reductase assay revealed significantly higher H_2_O_2_ and O_2_^−^ generation, respectively, on combined treatment with cocaine and Tat when compared with either treatment alone (Figure 2C-E). Specifically, the presence of catalase prevented the cocaine and Tat mediated increase in H_2_O_2_ production whereas no significant changes were observed in the presence of SOD. These findings indicate an additive increase in the ROS production by human pulmonary endothelial cells on exposure to both viral protein and cocaine.

### NADPH Oxidase is Involved in Cocaine and Tat Mediated ROS Generation

**Figure 3:**
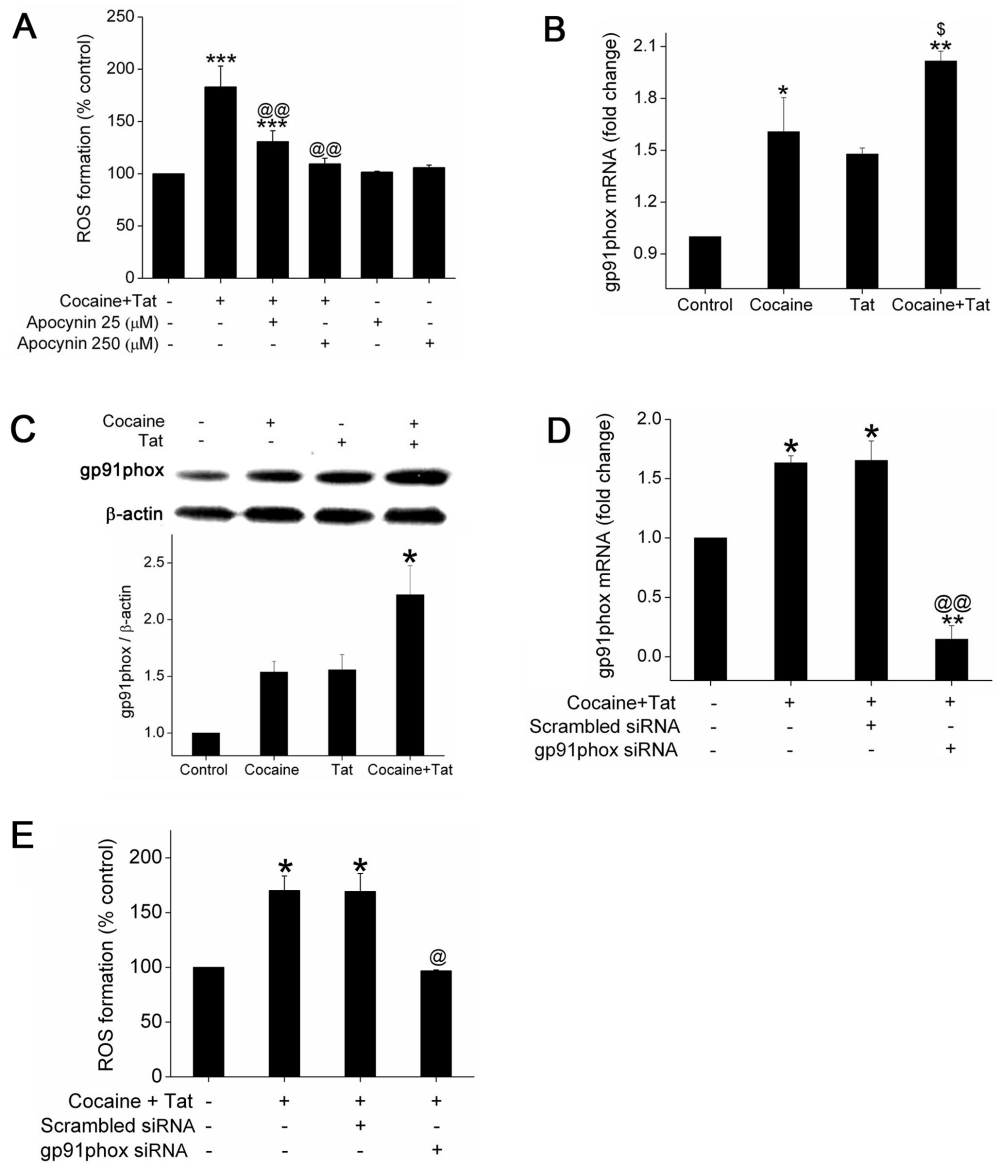
Inhibition or knockdown of NADPH oxidase attenuates Tat and cocaine-mediated ROS formation. (A) HPMECs were treated with various concentrations of apocynin for 30 min followed by exposure to Tat/cocaine for 1 hour. Total ROS formation was measured by DCFDA assay. (B) HPMECs were treated with cocaine and/or Tat for 24 h followed by quantitative mRNA analysis of gp91phox (NOX2) by Real-Time RT-PCR using the SYBR Green detection method. C) Western blot analysis of gp91phox in HPMECs treated with cocaine and/or Tat for 48 h.The upper panel is the representative blot of gp91phox expression and lower panel is the histogram showing the densitometry analyses of 3 independent experiments (mean±SEM). (D) NOX2 mRNA expression and E) ROS generation in HPMECs transfected with scrambled or gp-91^phox^ siRNA (5 nM). Cells were loaded with DCFDA for 30 min before 1 hour Tat/cocaine treatment for quantification of total ROS generation. The values shown are means (±SD) of two-three independent experiments. *P≤0.05, **P≤0.01, ***P≤0.001, compared to control; ^$^P<0.05 compared with Tat treatment alone, ^@^P≤0.05, ^@@^P≤0.001, compared to cocaine and Tat treatment.

To examine whether NADPH oxidase (NOX) is involved in generation of ROS in endothelial cells, we pretreated HPMEVCs with NOX inhibitor apocynin and measured ROS formation. As expected, apocynin reduced Tat and cocaine mediated ROS generation (Figure 3A). Since gp91phox (NOX2), a critical subunit of NOX, is the major source of ROS generation in vascular endothelial cells [24], we analyzed the mRNA expression of NOX2 on cocaine and/or Tat treatment. Interestingly, treatment of endothelial cells with either cocaine or Tat caused increase in gp91phox mRNA (Figure 3B) and protein (Figure 3C) expression compared with untreated cells. However, combined treatment with cocaine and Tat resulted in further increase in gp91phox mRNA and protein levels when compared with either cocaine or Tat treatment alone as shown in Figure 3B and 3C. To further confirm the role of NOX, ROS production was monitored in cells transfected with siRNA against NOX2. First the attenuated expression of gp91phox in siRNA transfected cells was confirmed by real time RT-PCR as illustrated in Figure 3D. Cells treated with cocaine and Tat, transfected with scrambled siRNA used as negative transfection control, also showed increase in NOX2 expression as was observed in un-transfected cells (Figure 3B). Furthermore, transfection of cells with siRNA gp91phox prevented the Tat and cocaine mediated increase in ROS generation (Figure 3E). Taken together, our data suggest a critical role of NOX in Tat and cocaine mediated ROS generation in pulmonary endothelial cells.

### ROS Dependent Enhanced Pulmonary Endothelial Disruption on Cocaine and Tat-treatment

**Figure 4:**
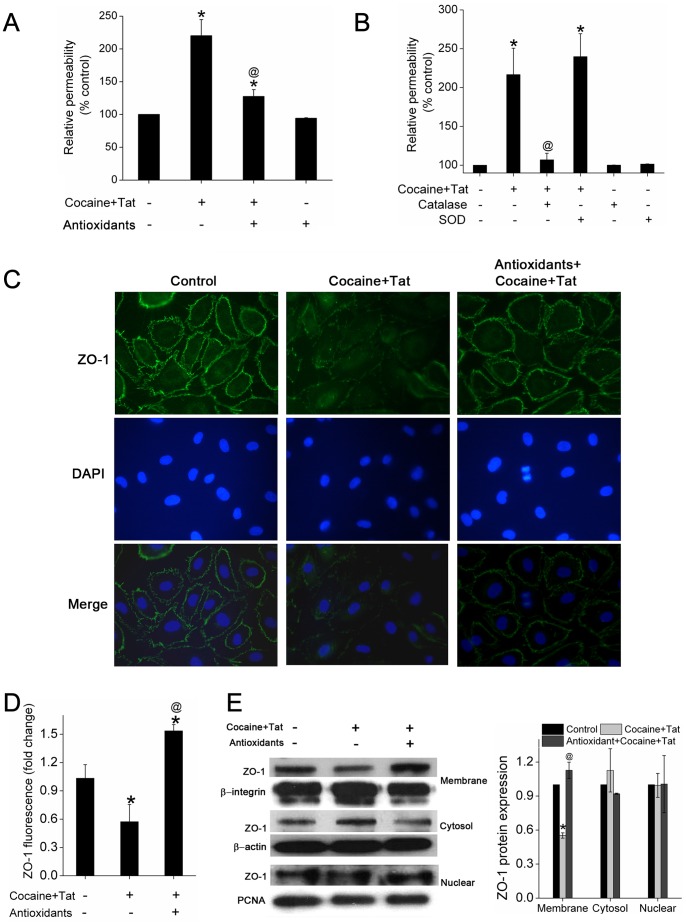
Antioxidant cocktail attenuates Tat and cocaine-mediated augmentation of endothelial dysfunction. Effect of antioxidants (A) and catalase or SOD (B) on cocaine (1 µM) and Tat (25 ng/ml) mediated barrier dysfunction of HPMECs. Confluent monolayers were grown on collagen-coated Transwell inserts and treated with antioxidant cocktail followed by Tat and cocaine exposure for 6 or 24 hours. Monolayers were then treated with FITC-dextran and after 15 min the fluorescence in the lower compartment was measured and expressed as percentage of basal fluorescence. (C) Effect of antioxidants on Tat and/or cocaine mediated down-regulation of tight junction protein expression in pulmonary arterial endothelial cells. Cells grown on coverslip were immunostained for TJP-1. (D) Quantification of ZO-1 immunofluorescence using ImageJ software. (E) Western blot analysis of ZO-1 in various cellular compartments. Blot is representative of at least three independent experiments with histogram showing the average densitometry analysis normalized to β-integrin for membrane fraction, β-actin for cytosolic fraction and PCNA for nuclear compartment. The values shown are means (±S.E.M.). *P≤0.001 compared to control; ^@^P≤0.001, compared to cocaine and Tat treatment.

We next examined if pre-treatment of cells with antioxidant cocktail could prevent the cocaine and Tat mediated ZO-1 disruption and endothelial dis-integrity. As shown in Figure 4, Tat and cocaine -mediated increase in monolayer permeability was reversed in the presence of antioxidant cocktail (Figure 4A). We next determined which type of ROS is important in the disassembly of HPMEC monolayer. Interestingly, inhibiting the levels of H_2_O_2_ with catalase prevented the cocaine and Tat mediated increase in monolayer permeability (Figure 4B) whereas pre-treatment of cells with SOD led to further increase in the cocaine and Tat mediated permeability. Therefore suggesting a critical role of H_2_O_2_ in cocaine and Tat mediated endothelial dysfunction.

As reported in our earlier findings, loss of ZO-1 expression at the periphery of the cocaine and Tat treated cells was significant as clearly observed in representative images (Figure 4C) and semi-quantitative analysis of immunofluorescence staining (Figure 4D). Meanwhile in the presence of antioxidants, ZO-1 remained localized along the cellular membrane as uniform and continuous structure in cocaine and Tat treated cells. Western blot analysis (Figure 4E) confirmed the loss of ZO-1 protein in the membrane fraction of cells treated with both Tat and cocaine whereas pre-treatment with antioxidants prevented this loss of ZO-1 expression in the membrane fraction. Therefore, our data indicate a causative role of ROS in Tat/cocaine-mediated disassembly of ZO-1 in HPMECs.

### Cocaine and Tat Mediated Activation of Ras/Raf/Erk Pathway is Involved in ZO-1 Disruption

**Figure 5:**
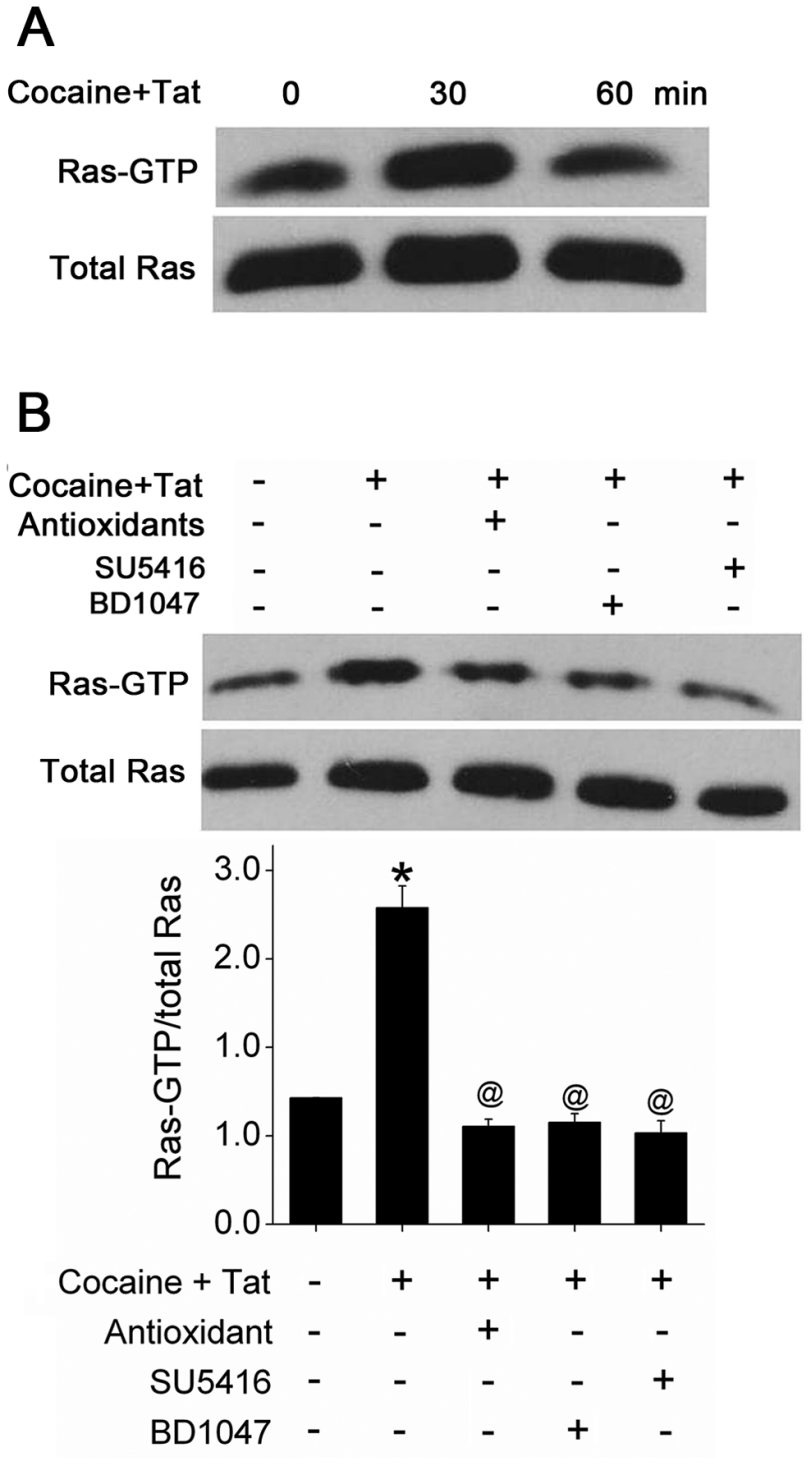
Activation of Ras/Raf/Erk pathway in Tat and cocaine exposed HPMECs. (A) Ras activation was assessed by pull-down assay in cells treated with Tat and cocaine for 30 or 60 min. (B) HPMECs were pre-treated with antioxidant cocktail, SU5416 or BD1047 for 5 min followed by Tat and cocaine treatment for 30 min. Representative western blot images are shown with histogram showing the average densitometry analysis of at least three independent experiments. Mean (±S.E.M.), *P≤0.001, compared to control; ^@^P≤0.001, compared to cocaine and Tat treatment.

**Figure 6:**
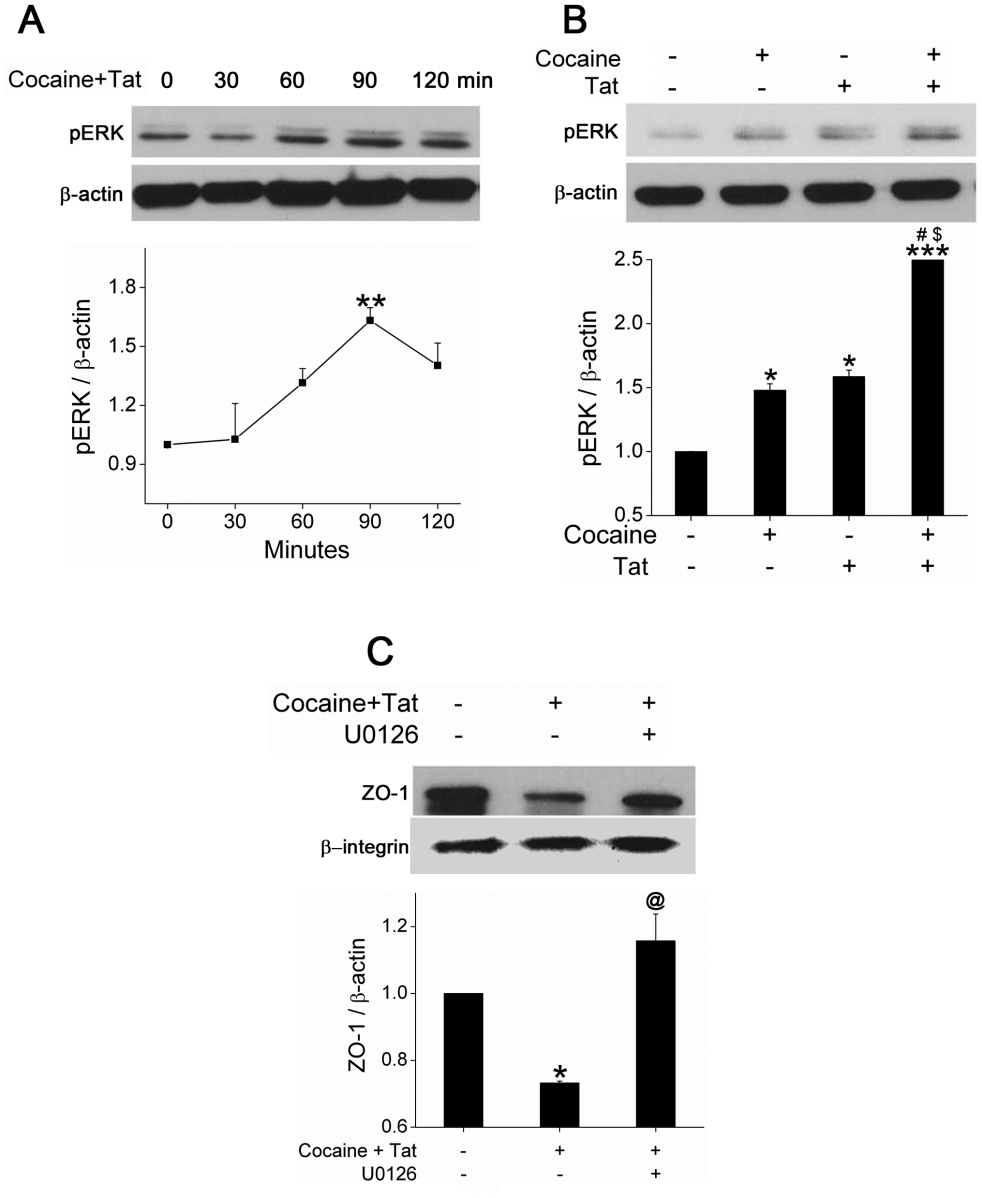
Blocking Tat and cocaine-mediated ERK activation reverses ZO-1 disruption in HPMECs. Phosphorylated ERK was detected by western blot analysis of HPMECs treated with (A) Tat and cocaine for different time intervals as indicated and (B) with cocaine and/or Tat for 1.5 hours. (C) ZO-1 expression analysis in HPMECs pre-treated with U0126 for 30 min followed by Tat and cocaine treatment for 24 hours. Membrane fraction was isolated using compartment protein fractionation kit and ZO-1 was detected by western blot analysis. Lower panels show the average densitometry analysis normalized to β-actin for total cellular extract (A and B) and β-integrin in case of membrane fraction (C) of at least three independent experiments. Mean (±S.E.M.). *P≤0.05, **P≤0.01, ***P≤0.001 compared to control; ^#^P≤0.001 compared to cocaine treatment. ^$^p<0.001 compared to Tat treatment, ^@^P≤0.01, compared to combined cocaine and Tat treatment.

To explore the potential mechanisms underlying Tat and cocaine-mediated ZO-1 disruption, we focused on redox regulated Ras/Raf/ERK signaling pathway [25]. Total cellular extract from cocaine and Tat treated HPMECs was used to perform Ras pull-down assay. As shown in Figure 5A, Ras was activated within 30 min of Tat and cocaine exposure. The activation was transient, dropping to basal level by 60 min. Pre-treatment with antioxidants, antagonist of VEGFR-2 or sigma receptor antagonist prevented Tat and cocaine-mediated Ras activation (Figure 5B). ERK_1/2_ MAP Kinase, downstream of Ras/Raf was found to be activated as early as 1 hour after cocaine and Tat treatment (Figure 6A). This increase in activation of ERK was significantly higher when compared Tat or cocaine treatment alone (Figure 6B) and pre-treatment of cells with U0126, an inhibitor of MEK_1/2_/ERK pathway reversed cocaine and Tat-mediated ZO-1 disruption (Figure 6C). Our data suggest that Tat and/or cocaine-induced ROS production contributes to activation of Ras/ERK signaling that leads to augmentation of ZO-1 disruption.

## Discussion

Our study offers *in-vitro* findings that HIV protein-Tat and cocaine disrupt tight junction protein, ZO-1 and induce related endothelial dysfunction via the ROS dependent Ras/ERK signaling pathway. We demonstrate that Tat and cocaine mediated ROS formation involves: sigma receptor and VEGFR-2 binding, is dependent on NADPH oxidase, and that the endothelial disruption this pathway confers can be mitigated with antioxidant treatment. Our findings provide new insights into the pathogenesis of HIV associated pulmonary arteriopathy as we describe the interplay of ROS and Ras/ERK signaling in Tat and cocaine mediated endothelial barrier dysfunction.

The mechanism for pulmonary arterial hypertension in HIV patients is elusive. However, animal studies revealed endothelial injury as an initial step in the development of pulmonary arteriopathy associated with PAH [8]. Recent studies demonstrate increased oxidative stress in patients with PAH suggesting its role in the pathogenesis of endothelial injury [26]. ROS is implicated as a mediator of endothelial injury as it is known to impair endothelial cell functions through direct cellular injury and/or by eliciting signal cascades [27]. The increased production of ROS [28], [29] and decrease in plasma antioxidant molecules seen in HIV-positive patients may explain in part the increased risk of developing PAH [30]. Evidence suggests that direct infection of HIV-1 within the pulmonary endothelium is not the source of increased ROS exposure within the pulmonary vascular bed. However, HIV-Tat is actively released by HIV-infected cells such as macrophages and T cells, and can have bystander effect on vascular endothelium. Our earlier study [31] and others [32] reveal that HIV proteins Tat or gp120 is associated with enhanced ROS formation.

Dynamic regulation of TJP function is fundamental to many physiological processes. Disruption of tight junctions drastically alters paracellular permeability and is a hallmark of many pathologic states. ROS induces rapid tyrosine phosphorylation and redistribution of TJPs leading to a decrease in trans-epithelial electrical resistance in endothelium [33] and subsequent disruption of pulmonary artery [34] or brain endothelial [35] [36] integrity. ZO-1 is a member of the membrane-associated guanylate kinases. It is involved in signal transduction as well as acts as scaffold to organize occludin at cell junction sites [37] and/or links occludin to actin cytoskeleton [38]. Thus, ZO-1 plays a regulatory role in cellular permeability [39], [40]. We previously reported synergistic loss of ZO-1 expression at the periphery of endothelial cells on combined exposure to cocaine and Tat [7] and literature suggests that ZO-1 is susceptible to ROS-mediated disruption [36], [41]. Here we report that levels of ROS, H_2_O_2_, and superoxide were significantly enhanced in Tat and cocaine combined treatment in comparison with either treatment alone. Importantly, pretreatment with an antioxidant cocktail prevented the cocaine and Tat mediated loss of ZO-1 from the membrane, however no significant alterations in ZO-1 expression was observed in the nuclear and cytosolic fractions of cocaine and Tat treated cells with or without antioxidant pre-treatment. Furthermore, antioxidant pre-treatment resulted in reduction in cocaine and Tat mediated enhanced monolayer permeability, suggesting Tat and cocaine induced ROS-generation plays an important role in tight junction protein disassembly and endothelium injury. Since catalase pre-treatment could completely prevent, whereas SOD pretreatment further enhanced the cocaine and Tat mediated increase in monolayer permeability, we speculate the involvement of H_2_O_2_ in this process. Given that both HIV-Tat and cocaine increase ROS, it is likely they may interact in concerted fashion to potentiate disruption of endothelium integrity.

HIV-Tat is known to bind VEGFR-2 (Flk-1/KDR) [15], [23], and VEGF is known to result in ROS production [42], [43] and cause endothelial damage [44]-[46]. In addition, cocaine is known to have high affinity for sigma-1 receptors. Binding results in translocation of sigma receptors to other areas of the cell including the plasma membrane where they are known to act as a molecular chaperone or signal modulator of other receptors or kinase(s) including Src family kinase (SFK) [47], that are known to be present in endothelial cells [48]. Activation of SFK is known to generate ROS [49] which in turn are known to activate SFK [50] and activate receptor tyrosine kinase(s) by phosphorylation [51]-[53]. We observed significant activation of Src following cocaine and Tat treatment of HPMECs with peak activation observed at 15 min post-treatment (data not shown). We found that antagonists of VEGFR-2 or sigma receptor prevented cocaine and Tat mediated additive increase in ROS generation, ZO-1 disruption, and endothelial dysfunction. Thus, simultaneous exposure of pulmonary endothelium with Tat and cocaine may induce a synergistic positive feedback loop between ROS and SFK, leading to enhanced activation of VEGFR-2 and resultant ROS production.

A variety of cellular signaling pathways, depending on the cell types and stimulus, have been identified to be involved in regulating TJPs. G-proteins [54], [55], protein kinase C [56], c-Src [57], Ca^2+^ [58], and c-AMP [59], [60] have been implicated in endothelium TJP biogenesis and pathology. Cellular signals stimulate VEGFR-2, a G-protein receptor and other cell surface receptors that localize in caveolae of endothelial cells leading to their hetero-trimerization and consequent activation of Ras [61]. Intracellular ROS functions as an important second messenger regulating the Ras/ERK signal transduction pathway [62]. The role of Ras in tight junction protein function has been explored in earlier studies in different experimental settings. Activation of Ras in Madin-Darby canine kidney cells (MDCK) epithelial cells is known to disrupt tight junction proteins including ZO-1 [63]. Our present study reveals that Tat and cocaine co-treatment increased Ras-GTP and an antioxidant cocktail partially blocked Ras activation, indicating the presence of oxidative stress and/or alterations of cellular redox status by Tat/cocaine may be responsible for Ras activation. The activation of Ras signaling was partially blocked by antagonists of VEGFR-2 or sigma receptor, suggesting the interplay between Tat/cocaine is responsible for redox- sensitive activation of Ras and subsequent ZO-1 disassembly. Therefore, Tat/cocaine-induced oxidative stress plays an important role in affecting endothelium integrity via activation of Ras pathway.

Furthermore, we observed significant activation of downstream effector of Ras, ERK1/2 kinase upon Tat and cocaine treatment. Pre-treatment of endothelial cells with MEK inhibitor U0126 that inhibits the activity of ERK1/2, prevented the Tat and cocaine mediated down-regulation of tight junction protein ZO-1 at the cell periphery, suggesting the role of ERK MAP kinase in ZO-1 disruption. Earlier reports suggest involvement of ERK, in Tat-mediated disruption of ZO-1 in mouse blood-brain barrier [64] or cytokines-induced tight junction protein disassembly in airway cells [65]. Enhanced ERK activity is associated with the disruption of TJP by H_2_O_2_ in endothelial cell monolayers [66] or by phorbol ester in corneal epithelium [67]. The exact mechanisms underlying Tat/cocaine mediated disruption of tight junction protein through Ras/ERK is not fully understood. However, it is likely that ERK activation may directly result in degradation, disruption, and rearrangement of ZO-1 [63], [67]. Additionally, ERK1/2 signaling pathway may activate NFκB, Jun/AP-1, and/or Elk which trans-activate inflammatory cytokines resulting in alterations of tight junction integrity and endothelial permeability [68], [69].

It is known that chronic cocaine exposure triggers endothelial damage seen in cocaine abusers [70], and our earlier studies reveal less tight junction proteins present in lung tissues of HIV patients with a history of IVDU than individuals with HIV infection or IVDU alone [7]. Based on the data presented here and in our previous reports, we speculate that HIV-Tat interacts with cocaine to generate reactive oxidative stress. The enhanced ROS formation in the presence of both Tat and cocaine elicit loss of endothelium integrity through redox sensitive Ras/ERK signaling pathway. Increased ROS is seen in HIV-infected individuals and cocaine users, thus interplay between HIV and cocaine establish an environment that induces endothelial injury and promotes arteriopathy which contributes to increased risk of developing HIV-PAH in individuals infected with HIV and a history of IVDU.
